# Dynamics and features of transmission clusters of HIV-1 subtypes in the state of São Paulo, Brazil

**DOI:** 10.3389/fpubh.2024.1384512

**Published:** 2024-06-05

**Authors:** Victor Pimentel, Andrea Pineda-Peña, Cruz S. Sebastião, João L. de Paula, Cintia M. Ahagon, Marta Pingarilho, M. Rosário O. Martins, Luana P. O. Coelho, Elaine M. Matsuda, Daniela Alves, Ana B. Abecasis, Luís F. M. Brígido

**Affiliations:** ^1^Global Health and Tropical Medicine, GHTM, Associate Laboratory in Translation and Innovation Towards Global Health, LA-REAL, Instituto de Higiene e Medicina Tropical (IHMT), Universidade NOVA de Lisboa (UNL), Lisbon, Portugal; ^2^Centro de Investigação em Saúde de Angola (CISA), Caxito, Angola; ^3^Instituto Nacional de Investigação em Saúde (INIS), Luanda, Angola; ^4^Instituto Adolfo Lutz, São Paulo, Brazil; ^5^Secretaria da Saúde de Santo André, São Paulo, Brazil

**Keywords:** HIV-1, genetic diversity, transmission clusters, São Paulo, Brazil

## Abstract

**Background:**

Molecular epidemiology techniques allow us to track the HIV-1 transmission dynamics. Herein, we combined genetic, clinical and epidemiological data collected during routine clinical treatment to evaluate the dynamics and characteristics of transmission clusters of the most prevalent HIV-1 subtypes in the state of São Paulo, Brazil.

**Methods:**

This was a cross-sectional study conducted with 2,518 persons living with HIV (PLWH) from 53 cities in São Paulo state between Jan 2004 to Feb 2015. The phylogenetic tree of protease/reverse transcriptase (PR/RT) regions was reconstructed by PhyML and ClusterPicker used to infer the transmission clusters based on Shimodaira–Hasegawa (SH) greater than 90% (phylogenetic support) and genetic distance less than 6%.

**Results:**

Of a total of 2,518 sequences, 2,260 were pure subtypes at the PR/RT region, being B (88%), F1 (8.1%), and C (4%). About 21.2% were naïve with a transmitted drug resistance (TDR) rate of 11.8%. A total of 414 (18.3%) of the sequences clustered. These clusters were less evident in subtype B (17.7%) and F1 (15.1%) than in subtype C (40.2%). Clustered sequences were from PLWH at least 5 years younger than non-clustered among subtypes B (*p* < 0.001) and C (*p* = 0.037). Men who have sex with men (MSM) predominated the cluster in subtype B (51%), C (85.7%), and F1 (63.6%; *p* < 0.05). The TDR rate in clustered patients was 15.4, 13.6, and 3.1% for subtypes B, F1, and C, respectively. Most of the infections in subtypes B (80%), C (64%), and F1 (59%) occurred within the state of São Paulo. The metropolitan area of São Paulo presented a high level of endogenous clustering for subtypes B and C. The São Paulo city had 46% endogenous clusters of subtype C.

**Conclusion:**

Our findings showed that MSM, antiretroviral therapy in Treatment-Naive (ART-naïve) patients, and HIV1-C, played an important role in the HIV epidemic in the São Paulo state. Further studies in transmission clusters are needed to guide the prevention intervention.

## 1 Introduction

São Paulo is the most populous State in Brazil and the driving force of the Brazilian economy, with an estimated population of 44,396,484 inhabitants in 2015 (21.6% of the Brazilian population and a third of the National grass product). The metropolitan area of São Paulo comprises 39 cities, and that of Campinas, 42 cities, corresponding to 47.8 and 9.6% of the state’s population, respectively.^1^ In São Paulo State, 236,434 AIDS cases were reported from 1980 to 2014. In 2015, a total of 1,137 new HIV infections were reported in São Paulo city (1).

HIV commonly spreads more rapidly in specific groups, such as those men who have sex with men (MSM), sex workers and people who inject drugs. According to the national epidemiological report, since 2008, the HIV epidemic has shown a significant increase in the number of cases among MSM. In the 18–24 age group, the proportion increased from 30.2% in 2007 to 43.7% in 2012 (2).

The HIV-1 epidemic in São Paulo is dynamic with a genetic complexity driven by subtypes B, F1 and recombinant BF1 (3–6), however recent studies have shown a continuous increase of subtype C among São Paulo patients (7, 8). The Brazilian government has been sponsoring a large-scale implementation of highly active antiretroviral therapy (HAART) since December 1996 and until June 2013, approximately 400,000 patients were receiving HAART in Brazil (9). The HAART improves the quality of life of the people living with HIV (PLWH) globally, however, virological failure when using HAART can lead to the emergence of drug resistance mutations (DRM) that increase the chance of new infections with resistant strains (10). Most studies conducted in Brazil have shown a transmitted drug resistance (TDR) prevalence similar to that observed among developed countries (11) and São Paulo follows this trend with around 8% of TDR reported (12).

Although several studies have been conducted in São Paulo State, the dynamics and determinants of transmission clusters of HIV-1 subtypes prevalent in the state of São Paulo remain unknown and deserve further investigation. In the present study, we combined clinical and epidemiological data in order to evaluate the dynamics and determinants of transmission clusters of the prevalent HIV-1 subtypes in the state of São Paulo, Brazil.

## 2 Materials and methods

### 2.1 Study design and setting

This was a cross-sectional study conducted with 2,518 PLWH from 53 cities in São Paulo state from 2004 to 2015. We included volunteers seeking genotype tests due to antiretroviral treatment (ART) failure and pre-treatment tests of newly diagnosed patients in two sites in the São Paulo metropolitan area. Blood samples were collected from consenting participants between Jan 2004 to Feb 2015 and sent to the Adolpho Lutz Institute (IAL) in São Paulo for a genotypic-resistance test. Moreover, a questionnaire was used to collect demographic, geographic, and clinical data [The CD4 T cell counts were obtained with Flow cytometry (BD, United States) and HIV plasma viral load was quantified using the contemporary methodology available at public HIV-1 viral load laboratory network including Branched DNA (Siemens, United States), NASBA (Nucleic Acid Sequence Based Amplification, United States) or real-time PCR (Abbott, United States)]. when available from each patient. Data was anonymized for these analyses, after reporting genotype results to clinical services to subsidize clinical decisions. The study was approved by the institutional ethical committee (CAAE 02298012.6.1001.0059).

### 2.2 HIV-1 sequencing, subtyping, and phylogenetic analysis

Partial HIV-1 *pol* sequences for blood samples were obtained by population sequencing using in-house protocols (12) or TruGene (Siemens, United States). The Pol amplicon encompassed the entire protease region and the first 230 amino acids of the reverse transcriptase, derived from 5 to 8 primers (both forward and reverse) to span the targeted sequence. For clinical applications, partial genomes were generated, and corresponding reports were sent to clinical services. However, for the purposes of this study, only sequences that met stringent quality control standards were included in the analysis The nucleotide sequences were subtyped using REGA v3.0 (13), Comet (14), SCUEL (15), and jPHMM (16). The sequences were submitted to the Los Alamos Quality Control tool, which can be accessed at the following URL.^2^ Sequences containing more than three frame-shift events or stop-codons were removed, along with those classified as hypermutated. We split the sequences, according to purity, into subtypes B, C and F1. We then selected 2,107 unique background control sequences by blasting our sequences against the Los Alamos database^3^ and the HIV database from Portugal consists of a repository of genetic sequences from the *pol* gene, obtained through antiretroviral resistance tests (PTHIVDB) conducted at Portugal’s main hospital, Centro Hospitalar Lisboa Ocidental (CHLO). For each subtype, we aligned our sequences against the 10 closest global background sequences selected with Blast using muscle.^4^ A preliminary phylogenetic analysis was done using a FastTree maximum-likelihood (ML) tree using the general time reversible (GTR) model nucleotide substitution (17). The codons associated with DRMs for surveillance were manually removed from the sequence alignment to prevent the possibility of clustering due to mutations selected by the current use of antiretroviral therapy. To analyze HIV-1 transmission dynamics, phylogenetic reconstructions were performed by ML criterion using RAxML version 7.4.8 (18) under the GTR + Γ model without partitions. Cluster Picker was used to define transmission clusters as those clusters with bootstrap support ≥90 and genetic distance ≥6.0% (19).

### 2.3 Geographic cluster dynamics inter and intra São Paulo state

Based on the origin of the outpatient service, clusters that contained ≥66% sequences with the same area from São Paulo State were classified as endogenous clusters. In the case of clusters with only two sequences, both must have been from the same locality to define an endogenous cluster. Outside clusters were referred to those with more than 34% of sequences from different areas.

### 2.4 HIV-1 drug resistance analysis

The partial HIV-1 *pol* sequences containing PR/RT regions were screened for the presence of DRMs using the Calibrated Population Resistance tool available,^5^ updated in 2016, according to the WHO’s Surveillance Drug Resistance Mutation (SDRM) 2009 list (20).

### 2.5 Statistical analysis

Descriptive statistics such as frequencies with percentages and medians with interquartile range (IQR) were used to summarize patients’ demographic and clinic characteristics. We use the Shapiro–Wilk test to check the normal distribution in order to choose parametric or non-parametric tests. Mann–Whitney U or Kruskal-Wallis nonparametric tests were used to compare two or more independent groups. Differences between proportions were calculated with the Chi-square test or Fisher’s exact test, as appropriate. We consider a significance level of 5% for all statistical analyses. The analysis was performed using SPSS v22 (IBM SPSS Statistics, United States).

## 3 Results

### 3.1 Demographic and clinical characteristics

A total of 2,518 eligible sequences were studied, most of the cases (71.6%) from the São Paulo metropolitan area. After sequence quality control, 25 (1.1%) of them were removed. All sequences with a recombinant signal were removed from the analysis (10.8%, 233/2518). Thus, 2,260 sequences from patients infected with subtypes B, C and F1 were kept for further analysis. Table 1 summarizes the demographic and clinical characteristics of the studied population. The median age of the 2,260 patients included was 38 (IQR: 30–46), of these, 65.1% were male. Heterosexuals (HET; 60.7%) and MSM (30.1%) were the most predominant. About 78.8% of patients were already on antiretroviral therapy and 21.2% were ART-naïve (with a TDR rate of around 11.8%). The median values of CD4 cell count and log10 viral load were 274 (IQR: 141–459) and 4.38 (IQR: 3.79–4.95), respectively. Regarding HIV-1 subtyping, subtype B (88.2%) was the most predominant, followed by subtypes F1 (8.19%) and subtype C (3.63%). Individuals infected by subtype C were 4 and 6 years younger than individuals infected by subtypes B and F1, respectively (*p* = 0.020). Statistically significant differences were also observed between the distribution of subtypes with treatment status (*p* < 0.001) and borderline for infection risk categories (*p* = 0.053). The differences in mean CD4+ T cell count values between subtypes B (268 cells/mm3), C (395 cells/mm3) and F1 (303 cells/mm3) were statistically significant (*p* = 0.004). On the other hand, although it was not statistically significant (*p* > 0.05), a high prevalence of TDR was observed in subtypes F1 (18.2%) and B (12.2%), while a lower prevalence was observed in subtype C (3.8%).

**Table 1:** Epidemiological characteristics and clinical data among HIV-1 positive patients from São Paulo by subtype.

Independent variables	*N* = 2,260	Subtype B (*N* = 1993, 88.2%)	Subtype C (*N* = 82, 3.63%)	Subtype F1 (*N* = 185, 8.19%)	*p*-value
Age median (IQR)	38 (30–46)	38 (30–46)	34 (28–42)	40 (31–45)	**0.020**
Gender, n (%)					
Male	1,392 (65.1)	1,248 (65.8)	53 (71.6)	91 (53.8)	**0.004**
Female	747 (34.9)	648 (34.2)	21 (28.4)	78 (46.2)	
Risk Factor, n (%)					
HET	753 (60.7)	688 (61.9)	24 (41.4)	41 (56.9)	0.053*
MSM	373 (30.1)	319 (28.7)	34 (58.6)	20 (27.8)	
PWID	17 (1.4)	17 (1.5)	0 (0.0)	0 (0.0)	
MTCT	98 (7.9)	87 (7.8)	0 (0.0)	11 (15.3)	
Therapy status, n (%)					
Naïve	427 (21.2)	353 (19.7)	52 (68.4)	22 (14.3)	**<0.001**
Treated	1,591 (78.8)	1,435 (80.3)	24 (31.6)	132 (85.7)	
TDR, n (%)	49 (11.8)	43 (12.2)	2 (3.8)	4 (18.2)	0.982
CD4 median (IQR)	274 (141–459)	268 (136–452)	395 (204–577)	303 (175–504)	**0.004**
VL (log_10_) median (IQR)	4.38 (3.79–4.95)	4.30 (3.79–4.94)	4.45 (3.69–5.20)	4.39 (3.70–4.98)	0.716

IQR, Interquartile range; HET, heterosexual; MSM, men who have sex with men; PWID, persons who inject drugs; MTCT, Mother-to-child transmission; VL, Viral Load (Log10). Bold numbers mean that results were statistically significant to the Kruskal Wallis test or Chi-square or the alternative *Fisher exact test (*p* < 0.05).

### 3.2 Demographic and clinical profile of ART-naïve and experienced patients

The characteristics related to ART-naive and experienced patients are described in Table 2. ART-naive patients were 10 years younger than ART-experienced patients in subtypes B or C and 6 years younger in subtype F1. The distribution of sex between ART-naïve and treated patients was statistically significant within subtype B (*p* < 0.001) and C (*p* = 0.020). The transmission route depending on the treatment status (naïve vs. treated) was significant in all subtypes, whether B, C or F1 (*p* < 0.05). MSM patients predominated the naïve population, whether those infected with subtype B (74%), subtype C (71.7%) and F1 (57.1%; *p* < 0.05). The CD4+ T cell count in ART-naïve patients (ranging from 464 to 545 cells/mm3) was higher than that observed in ART-experienced patients (ranging from 204 to 285 cells/mm3), regardless of HIV-1 subtypes (*p* < 0.05).

**Table 2:** Epidemiological data of HIV-1 positive patients from São Paulo by subtype according to therapy.

Independent variables	Subtype B (*N* = 1788)				Subtype C (*N* = 82)				Subtype F1 (F1 = 185)			
	Naïve (*N* = 353)	Treated (*N* = 1,435)	Total	*p*-value	Naïve (*N* = 52)	Treated (*N* = 24)	Total	*p*-value	Naïve (*N* = 22)	Treated (*N* = 124)	Total	*p*-value
Age, median (IQR)	30 (25–38)	40 (32–47)	38 (30–46)	**<0.001**	31.5 (28–38.5)	40 (31–46)	34 (28–42)	**0.012**	34 (26–47.5)	40.5 (33–45)	34 (28–42)	0.334
Sex, n (%)												
Male	292 (83.7)	864 (62.1)	1,156 (66.4)	**<0.001**	41 (80.4)	10 (52.6)	51 (72.9)	**0.020**	15 (68.2)	64 (51.6)	79 (54.1)	0.151
Female	57 (16.3)	528 (37.9)	585 (33.6)		10 (19.6)	9 (47.4)	19 (27.1)		7 (31.8)	60 (48.4)	67 (45.9)	
Transmission route, n (%)												
HET	74 (23.2)	550 (76.6)	624 (60.2)	**<0.001**	13 (28.3)	9 (90.0)	22 (39.3)	**<0.001**	8 (38.1)	26 (60.5)	34 (53.1)	**0.002**
MSM	236 (74)	73 (10.2)	309 (29.8)		33 (71.7)	1 (10.0)	34 (60.7)		12 (57.1)	7 (16.3)	19 (29.7)	
PWID	1 (0.3)	16 (2.2)	17 (1.6)		0 (0.0)	0 (0.0)	0 (0.0)		0 (0.0)	0 (0.0)	0 (0.0)	
MTCT	8 (2.5)	79 (11)	87 (8.4)		0 (0.0)	0 (0.0)	0 (0.0)		1 (4.8)	10 (23.3)	11 (17.2)	
DRMs, n (%)	43 (12.2)	1,260 (87.8)	1,303(72.9)	**<0.001**	2 (3.8)	20 (83.3)	22 (28.9)	**<0.001**	4 (18.2)	113 (85.6)	117 (76)	**<0.001**
CD4, median (IQR)	464 (324–642)	241 (117–404)	268 (136–452)	**<0.001**	545 (400.2–703)	204 (106–274)	395 (204–577)	**<0.001**	526 (282–673.7)	285 (157–454)	395 (204–577)	**0.003**
VL (log_10_), median (IQR)	4.39 (3.18–4.89)	4.40 (3.82–4.95)	4.37 (3.79–4.94)	0.874	4.31 (3.45–5.22)	4.52 (4.08–5.21)	4.45 (3.69–5.20)	0.820	4.42 (3.57–5.3)	4.5 (3.72–4.9)	4.45 (3.69–5.2)	0.899

IQR, Interquartile range; HET, heterosexual; MSM, men who have sex with men; PWID, persons who inject drugs; MTCT, Mother-to-child transmission; VL, Viral Load (Log10). Bold numbers mean that results were statistically significant to the Kruskal Wallis test or Chi-square or the alternative *Fisher exact test (*p* < 0.05).

### 3.3 Features of transmission clusters of HIV-1 subtypes

The dynamics and demographic characteristics related to HIV-1 subtype transmission clusters are presented in Table 3 and Supplementary Data S1–S3. About 18.3% (414/2260) of the sequences clustered with one or more other sequences. This clustering was less pronounced in subtypes B (17.7%) and F1 (15.1), compared to C (40.2%). The median age was significantly associated with clustering for subtypes B (*p* < 0.001) and C (*p* = 0.037). The median age of clustered individuals for subtype B (33 years, IQR:26–41) was 6 years younger than the non-clustered individuals (39 years, IQR: 31–47). In subtype C, the median age of clustered individuals (30 years, IQR: 27–32) was 5 years younger than the non-clustered group (35 years, IQR, 30–45). The clustering of patients was related to sex, in patients infected with subtype B (*p* = 0.001) and C (*p* = 0.012). Within individuals in the transmission cluster, the proportion of males was higher for subtypes B (73.7%, *p* = 0.001), C (71.6%, *p* = 0.012) and F1 (68%, albeit without no statistical significance observed, *p* = 0.124). Among the subtype B clusters, 51% were MSM, 41.4% HET, 6% mother-to-child transmission (MTCT), and 1.3% persons who inject drugs (PWID; *p* < 0.001). In subtype C, 85.7% of the clusters were composed of MSM patients and 14.3% HET (*p* < 0.001). Within subtype F1 clusters, 63.6% were MSM and 36.4% HET (*p* = 0.011). Dyads clusters were frequently observed in patients grouped with subtype B (68%) and F1 (53.6%) while patients grouped with subtype C presented clusters with 3 or more sequences. The TDR rate in clustered patients from subtype B was 15.4%, for subtype C was 3.1% and subtype F1 was 13.6%, although no statistical difference was observed when compared to non-clustered patients of the same HIV-1 subtypes (*p* > 0.05). The difference in CD4+ T cell counts was significant, comparing clustered to non-clustered patients only from subtype C (*p* = 0.003).

**Table 3:** Epidemiological and clinical data of HIV-1 positive patients based on transmission clusters (*N* = 414) by subtype.

Independent variables	Subtype B (*N* = 1993)				Subtype C (*N* = 82)				Subtype F1 (*N* = 185)			
	Non-cluster (*N* = 1,640)	Cluster (*N* = 353, 17.7%)	Total (*N* = 1993)	*p*-value	Non-cluster (*N* = 49)	Cluster (*N* = 33, 40.2%)	Total (*N* = 82)	*p*-value	Non-cluster (*N* = 157)	Cluster (*N* = 28, 15.1%)	Total (*N* = 185)	*p*-value
Age median (IQR)	39 (31–47)	33 (26–41)	38 (30–46)	**<0.001**	35 (30–45)	30 (27–37)	34 (28–42)	**0.037**	40 (31–45)	39.5 (29–44.)	40 (31–45)	0.987
Gender, n (%)												
Male	1,002 (64.1)	246 (73.7)	1,248 (65.8)	**0.001**	26 (60.5)	27 (87.1)	53 (71.6)	**0.012**	74 (51.4)	17 (68)	91 (53.8)	0.124
Female	560 (35.9)	88 (26.3)	648 (34.2)		17 (39.5)	4 (12.9)	21 (28.4)		70 (48.6)	8 (32)	78 (46.2)	
Risk Factor, n (%)												
HET	592 (67.3)	96 (41.4)	688 (61.9)	**<0.001**	20 (66.7)	4 (14.3)	24 (41.4)	**<0.001**	37 (60.7)	4 (36.4)	41 (56.9)	0.011*
MSM	200 (22.8)	119 (51.3)	319 (28.7)		10 (33.3)	24 (85.7)	34 (58.6)		13 (21.3)	7 (63.6)	20 (27.8)	
PWID	14 (1.6)	3 (1.3)	17 (1.5)		0 (0)	0 (0)	0 (0)		0 (0)	0 (0)	0 (0)	
MTCT	73 (8.3)	14 (6.0)	87 (7.8)		0 (0)	0 (0)	0 (0)		11 (18)	0 (0)	11 (15.3)	
Type of cluster, n (%)												
Dyads clusters	-	240 (68)	240 (68)	-	-	16 (48.5)	16 (48.5)	-	-	15 (53.6)	15 (53.6)	-
**≥**3 sequences	-	113 (32)	113 (32)	-	-	17 (51.5)	17 (51.5)	-	-	13 (46.4)	13 (46.4)	-
TDR, n (%)	22 (10.0)	21 (15.4)	43 (12.2)	0.129	1 (2.3)	1 (3.1)	2 (2.4)	1.000*	1 (7.1)	3 (13.6)	4 (18.2)	0.427*
CD4 median (IQR)	263 (131–446)	299 (153–516)	268 (136–452)	0.392	262 (153–448)	549 (371–697)	395 (204–577)	**0.003**	294 (170–500)	318 (194–537)	303 (175–504)	0.748
VL median (IQR)	4.37 (3.79–4.92)	4.39 (3.81–4.99)	4.30 (3.79–4.94)	0.372	4.31 (3.45–5.22)	4.52 (4.08–5.21)	4.45 (3.69–5.20)	0.254	4.39 (3.72–4.98)	4.42 (3.57–4.99)	4.39 (3.70–4.98)	0.940

IQR, Interquartile range; HET, heterosexual; MSM, men who have sex with men; PWID, persons who inject drugs; MTCT, Mother-to-child transmission; VL, Viral Load (Log10). Bold numbers mean that results were statistically significant to the Kruskal Wallis test or Chi-square or the alternative *Fisher exact test (*p* < 0.05).

### 3.4 Clustering dynamics of HIV-1 subtypes

**Figure 1:**
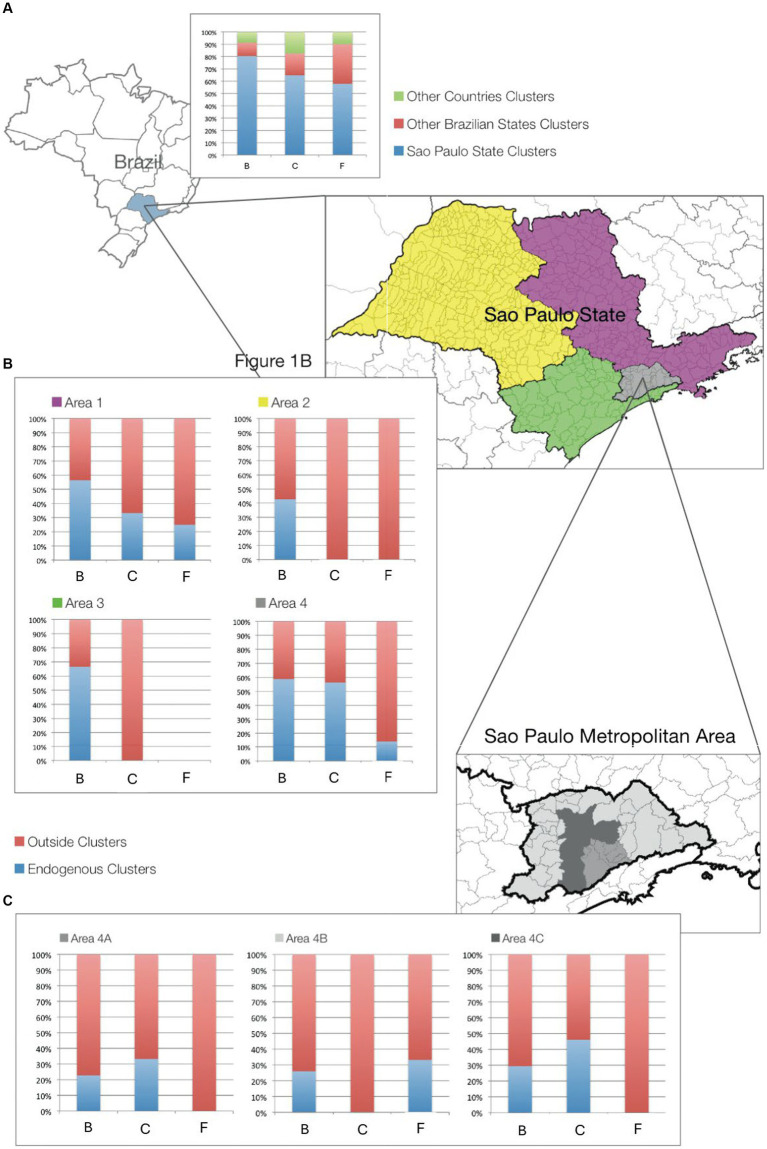
Types of Geographic clusters identified in patients from São Paulo State. The figure depicts cluster composition, by HIV-1 circulating subtype, according to the origin of sequences that compose the cluster as (i) Endogenous cluster (Blue bars) - cluster that includes at least 66% of its compounding members from the same geographical location, (ii) outside clusters, red bars, for clusters that are comprised of less than 66% of local cases. **(1A)** Further divides the outside clusters as clustering with foreign sequences (orange bar), from other countries, and those with only national (other states) clusters (red). **(B)** Shows the geographic division used to analyze the São Paulo State transmission cluster into 4 smaller areas. The zone highlighted in purple represents Area 1, yellow represents Area 2, the green zone represents Area 3 and the gray zone represents Area 4. **(C)** Zoom in São Paulo metropolitan area (zone 4). Light gray represents the periphery region of the metropolitan area, intermediate gray represents the major ABCD cities and dark gray represents São Paulo city.

The dynamics of HIV-1 subtype clustering are shown in Figure 1. It was observed that about 80% of subtype B infections occurred within the state of São Paulo, while 10% were associated with other Brazilian states and 8% with sequences from other countries. For subtype C, we found that 64% of transmissions occurred within the state of São Paulo while 18% were related to clusters of other Brazilian states and 18% clusters from other countries. In subtype F1, 59% of transmissions occurred within the state, 30% occurred in other Brazilian states and 11% were associated with other countries (Figure 1A). To better understand micro-epidemics in the state of São Paulo, we divided the map of the state according to the geographic location, gathering nearby municipalities. We found that areas 1 (Northeast, NE) and 4 (Metropolitan, Metro), where most sequences originated, have a similar profile, with approximately 60% of subtype B of endogenous clusters, suggesting transmissions within these regions. For the subtype C in the area NE, about 30% of the transmissions occurred within this location, while in the Metropolitan area over 50% occurred within the area. The subtype F1 had a low presence of endogenous clusters in the aforementioned areas (1 and 4). Regarding areas 2 (Northwest, NW) and 3 (South, SO). Subtype B show a similar profile of about half of the sequences showing endogenous clusters but without the identification of endogenous clusters from subtypes C and F1 available sequences (Figure 1B). The metropolitan area of São Paulo contributed with the larger number of samples and represents the most important region within the state. We therefore further divided it into three sub-areas (São Paulo city, ABCD region and Periphery). The peripheral region of the Greater São Paulo (4A), showed a different profile for the origin of transmission clusters, compared to regions 4B and 4C. We find in this highly populated area a high level of endogenous clustering for subtypes B and C. It is noteworthy that the region of São Paulo city had about 46% of endogenous clusters of subtype C. Interestingly, the periphery region is responsible for about 33% of endogenous clustering of subtype F1, similar to that observed in area NE (Figure 1C).

## 4 Discussion

To improve the knowledge of the HIV-1 transmission dynamics in the state of São Paulo, Brazil, this study considered a large dataset that examines the demographic and clinical characteristics of individuals infected by the subtypes B, C and F1. The observed prevalence of HIV-1 subtypes was similar to that observed in previous studies in the region being the epidemic was driven by subtype B, followed by subtype F and the less frequent, subtype C (21). However, when only patients with recent diagnoses (between January 2013 and February 2015) are considered, the frequency of subtype C is about 10%. This picture contrasts with that of the early 2000s when the prevalence of the subtype C in the region was approximately 3.8% (4). This proportion is consistent with more recent studies on HIV-1 diversity, which report an increase in the subtype C in São Paulo (8, 22). Another remarkable concern involving subtype C is the high proportion of ART-naïve patients compared to ART-experienced patients, supporting the hypothesis of a more recent introduction of subtype C in the state of São Paulo (23).

Our findings showed that subtype B had a lower median CD4 T-cell count compared to subtypes F1 and C, as also recorded in previous studies (24), however, that comparison revealed no differences in viral load levels between the HIV-1 subtypes. This study has not performed any survival analyses on the three subtypes, though other authors have suggested an association between the viral subtype and progression to AIDS (25–28).

This study includes samples from 53 different cities of São Paulo state which is responsible for 32% of the Brazilian AIDS epidemic. Surveillance studies on primary resistance performed in Brazil estimated TDR prevalence between 5 to 15%, depending on the geographic region (12, 29, 30). Another study conducted in 2015 detected a primary resistance rate of 9.2% in samples from patients with recent diagnoses in São Paulo state (31). The current study found 11.8% of TDR, with a high rate of resistance observed in subtypes F1 (18.2%), followed by subtypes B (12.2%) and C (3.8%). Previous studies conducted in the South of Brazil and involving a cohort of ART-naïve patients found a lower rate of DRM associated with resistance to subtype C compared to subtype B (32). Though many studies performed in different Brazilian states have reported rates of primary resistance, there is still a lack of information on this rate among different high-risk groups. It is known that, in Brazil, the MSM population is 29 times more susceptible to being infected compared to the whole population (33). In Latin America, MSM is the most commonly exposed to the risk of HIV-1 infection and represents the highest portion of new infections (34). The study by Bermúdez-Aza et al. (35), evaluated 299 HIV-infected MSM from 9 Brazilian cities and reported a rate of primary resistance of around 19.3% which was consistent with the data observed in the present study.

The concept of transmission clusters is controversial, and consensus has yet to be established. The definition of clusters adopted in this study was determined by bootstrap support ≥90% and a genetic distance ≤6.0%. Though many studies have adopted a genetic distance of up to 4.5%, we believe that the threshold adopted is necessarily more flexible, given the fact that our study considers 10 years of the epidemic, during which time patients studied have been in different phases of the disease and with different time of within-host viral evolution. Therefore, our study detected a putative low cluster prevalence of 18.3%. This low cluster prevalence could be explained in part because we were conservative when defining the cluster using two approaches, genetic distance and branch support. We found statistical differences in the clustering when the three circulating subtypes were evaluated. In subtype C, 40% of the patients were found to be transmission clusters, a value much higher proportion than that found among the B and F1 subtypes. One hypothesis for this difference is the fact that subtype C was introduced more recently and is affected by the distance from the founder virus (36, 37).

Studies using phylogenetics to describe transmission clusters are being increasingly used in developed countries. The study by Lewis et al. (38) describes the HIV-1 transmission cluster in London where was reported that approximately 25% of the MSM population was associated with various clusters. Meanwhile, a study involving a heterosexual cohort in the United Kingdom reported that only 5% of the population studied was associated with transmission clusters (39).

Based on our analysis of transmission clusters, it can be determined that, regardless of the viral subtype, clusters are most represented by the MSM and ART-naïve patients that are driving the epidemic in São Paulo state. The individual risk factors experienced by MSM have been well documented. They include high frequencies of different partners, unprotected sex, and high-index viral loads (40, 41). Another factor involved in MSM is the peculiar risk associated with anal sex, as the behaviors associated with it, increase the efficacy of viral transmission, resulting in a probability of HIV transmission via anal sex is 18 times higher than vaginal sex (42).

Another interesting finding was that patients with primary resistance to subtypes B and F1 had higher associations with transmission clusters. Indeed, many individuals carrying the virus with TDR, even after removing the TDR codons, were linked to other patients, a finding that suggests domestication or endogenic transmission events. Paradoxically, the mutations associated with resistance that prevail in these clusters do not seem to reduce viral fitness highlighting the importance of pre-treatment genotype testing among recently diagnosed individuals when NNRTI-based regimens are being considered. In this line, a study involving a Swiss cohort found that ART-naïve individuals are most commonly responsible for the transmission of resistant strains in the MSM (43).

We observed that of the HIV epidemic in the state of São Paulo, 81% of the clusters in subtype B were found to be endogenous, 11% originated in other states, and 8% were from other countries. This finding may be explained by the relatively early introduction of this subtype in Brazil, which occurred in the 1960s (44) as a result, the epidemic seems to be self-sustained. Among the subtypes studied, subtype C was found to be the most frequent in the cluster originating outside of Brazil; the clusters originating in southern Brazilian states were also found to have an important influence. This finding may also be explained by the recent introduction of subtype C into the state, as well as by the fact that this is the most prevalent subtype in the world (45). Other analyses also support the origin of the subtype C in São Paulo from cities in the south of Brazil (23). Subtype F1 was found to have an important portion of clusters originating in other Brazilian states and a low percentage of clusters from outside of Brazil, perhaps because of its low prevalence worldwide.

To further clarify the issue of transmission cluster origins within the state, we divided the state into four geopolitical regions. This division revealed that Regions Northeast and the Metropolitan area have very similar profiles. They reflected independent epidemics in which more than 50% of the subtype B clusters had local origins. These differences are likely caused by the fact that each of these regions possesses its independent economic centers and a relatively mature, even though concentrated epidemic. Subtype C was found to have an important component of endogenous clusters, particularly within the metropolitan area, and was also found to be associated with the MSM population. This finding is partially similar to reports in the South of Brazil, where the epidemic linked to this subtype is associated with both risk groups, the MSM and heterosexuals (46). However, the Northwest and South regions differed from the others described above in that only subtype B was found to have an important proportion of endogenous clusters. This difference may be due to the underrepresentation of samples from the regions and/or to the low prevalence of non-B subtypes in this geographic region. However, we evaluated a large dataset from most of the regions analyzed. We take into account the reported number of HIV infections by geographic location. Thus, the percentage of HIV-1 infected patients by location follows the distribution: area 1 (Northeast, NE; 0.78%), area 2 (Northwest, NW; 0.72%), area 3 (SOUTH, SO; 0.4%) and area 4 (Metropolitan, Metro; 1.4%). Highlighting that the area with the lowest representation (area 3), which has its representation ranging from 0.4 to 0.7% if we exclude the city of Santos, which for logistical issues was not represented in our sample cohort.

Within the state of São Paulo, we found independent micro-epidemics, the strongest of which involve subtype B. We further subdivided the São Paulo metropolitan area, with a population of over 14 million into 3 different sub-regions in an attempt to understand the role of each region in the local epidemic. One particularly noteworthy finding was the fact that there were only endogenous clusters of subtype C in Region 4a (the ABCD region) and in Region 4c (the São Paulo city). It is very likely that this subtype was introduced into these two sub-regions independently and then spread independently. Meanwhile, the subtype F1 epidemic is largely confined to the periphery of the metropolitan area, among individuals in the neighborhood of the region (region 4b) and is likely to have spread from there to other areas within the city or the opposite but maintained a local spread.

We recognize the limitations of our study and a potential bias in the results. The most important of which is the sample bias, the data sampling method used, and the disproportionate amount of samples from the different cities within the state. Our research utilized sequences from the partial pol region of the HIV genome, which inherently restricts the assessment of phylogenetic signals and particularly impacts the identification of recombination events in other genomic regions. We also realize that phylogeographic and migration analyses could give us further insight into the concern of intra-city transmission. On the other hand, this study was performed based on genotyping services open to all public clinics in the State. Albeit limitations in sample transportation may have diminished the amount of samples from areas farther away from the laboratory, it probably did not impact the subtype distribution and other potential inclusion bias.

## 5 Conclusion

Our data suggests that the epidemic in the state of São Paulo is driven by the MSM population with a higher level of TDR, regardless of the HIV-1 subtype, and that the epidemic is sustained by ART-naïve patients. The prevalence of subtype C in the epidemic is growing in the state, particularly in the greater São Paulo metropolitan area, where individuals with subtype C infection are twice as likely to be in transmission cluster than those infected with subtypes B and F1.

## Data availability statement

The authors acknowledge that the data presented in this study must be deposited and made publicly available in an acceptable repository, prior to publication. Frontiers cannot accept a manuscript that does not adhere to our open data policies.

## Ethics statement

The studies involving humans were approved by The study was approved by the institutional ethical committee (CAAE 02298012.6.1001.0059). The studies were conducted in accordance with the local legislation and institutional requirements. The participants provided their written informed consent to participate in this study.

## Author contributions

VP: Data curation, Formal analysis, Investigation, Methodology, Supervision, Validation, Writing – original draft, Writing – review & editing. AP-P: Data curation, Formal analysis, Validation, Visualization, Writing – original draft, Writing – review & editing. CS: Data curation, Formal analysis, Validation, Visualization, Writing – original draft, Writing – review & editing. JP: Data curation, Methodology, Writing – original draft, Writing – review & editing. CA: Data curation, Methodology, Writing – original draft, Writing – review & editing. MP: Data curation, Methodology, Writing – original draft, Writing – review & editing. MM: Data curation, Writing – review & editing, Writing – original draft. LC: Data curation, Methodology, Writing – original draft, Writing – review & editing. EM: Data curation, Writing – review & editing, Writing – original draft. DA: Data curation, Methodology, Writing – review & editing, Writing – original draft. AA: Conceptualization, Data curation, Methodology, Resources, Supervision, Validation, Writing – original draft, Writing – review & editing. LB: Conceptualization, Methodology, Project administration, Resources, Supervision, Validation, Writing – original draft, Writing – review & editing.

## Footnotes

^1^ https://www.ibge.gov.br/explica/pib.php

^2^ https://www.hiv.lanl.gov

^3^ https://www.hiv.lanl.gov/content/index

^4^ http://www.drive5.com/muscle

^5^ https://hivdb.stanford.edu/cpr/form/PRRT/v8.1
