# Supplementary material for: Dynamics and features of transmission clusters of HIV-1 subtypes in the state of São Paulo, Brazil
Source: Front Public Health. 2024 Jun 5;12:1384512. doi: 10.3389/fpubh.2024.1384512 (PMC11187794; doi:10.3389/fpubh.2024.1384512)
Supplement: Supplementary file 1 [file Data_Sheet_1.PDF]

Dynamics and features of transmission clusters of HIV-1 subtypes in the state of Sao Paulo, Brazil

Victor Pimentel[1,*], Andrea Pineda-Peña[1], Cruz S. Sebastião[1,2,3], João L. de Paula[4], Cintia M. Ahagon[4], Marta Pingarilho[1], M Rosário O Martins[1], Luana P. O. Coelho[4], Elaine M. Matsuda[4,5], Daniela Alves[1], Ana B. Abecasis[1,#], Luís F.M. Brígido[4,#]

[1] Global Health and Tropical Medicine, GHTM, Associate Laboratory in Translation and Innovation Towards Global Health, LA-REAL, Instituto de Higiene e Medicina Tropical (IHMT), Universidade NOVA de Lisboa (UNL), Lisboa, Portugal
[2] Centro de Investigação em Saúde de Angola (CISA), Caxito, Angola
[3] Instituto Nacional de Investigação em Saúde (INIS), Luanda, Angola
[4] Instituto Adolfo Lutz, São Paulo, Brasil
[5] Secretaria da Saúde de Santo André, São Paulo, Brasil

# These authors contributed equally to this work.
* Corresponding author: victor.pimentel@ihmt.unl.pt

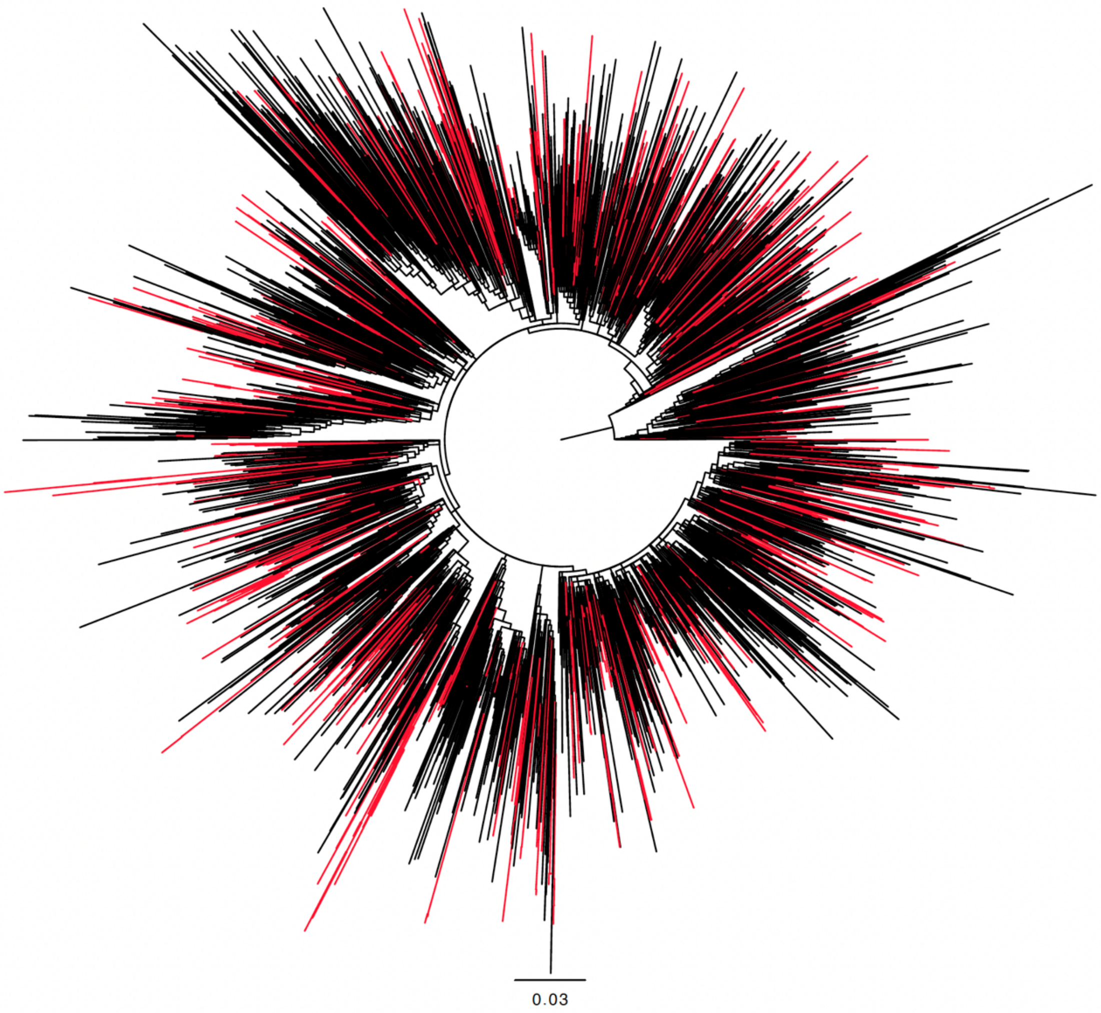

Figure S1. Phylogenetic analysis corresponding to the pol gene of 3,708 sequences of subtype B of HIV-1. The sequences were aligned against global control sequences from the BLAST collected from the Los Alamos database. Three reference sequences of pure subtype C were used as an outgroup. Bootstrap values equal to or higher than 90% and genetic distance less than 0.06 are represented in the tree as clusters marked in red. The scale bar indicates 3% divergence in the nucleotide sequence.

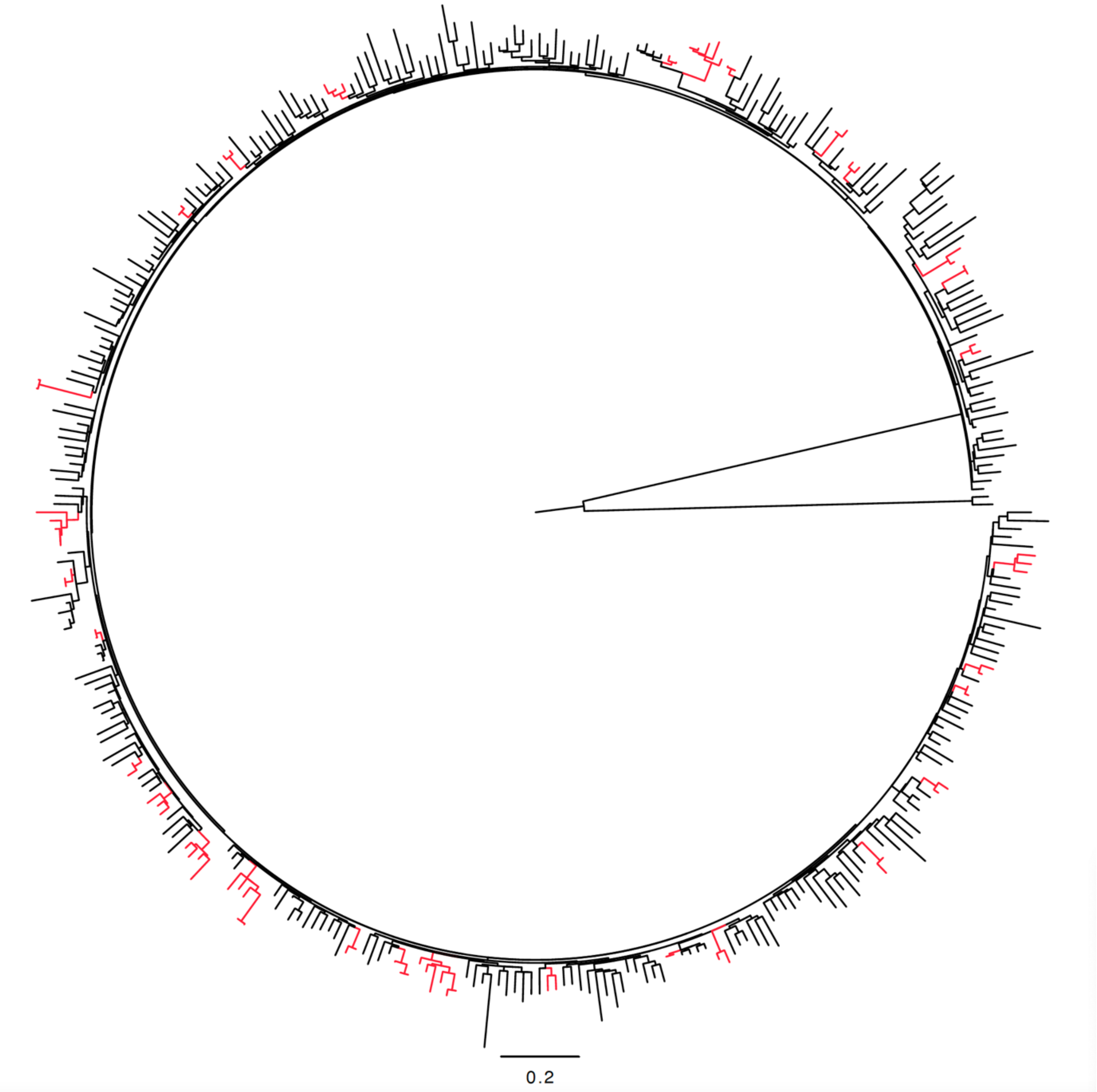

Figure S2. Phylogenetic analysis corresponding to the pol gene of 359 sequences of subtype F1 of HIV-1. The sequences were aligned against global control sequences from the BLAST collected from the Los Alamos database. Two reference sequences of pure subtype B were used as an outgroup. Bootstrap values equal to or higher than 90% and genetic distance less than 0.06 are represented in the tree as clusters marked in red. The scale bar indicates 2% divergence in the nucleotide sequence.

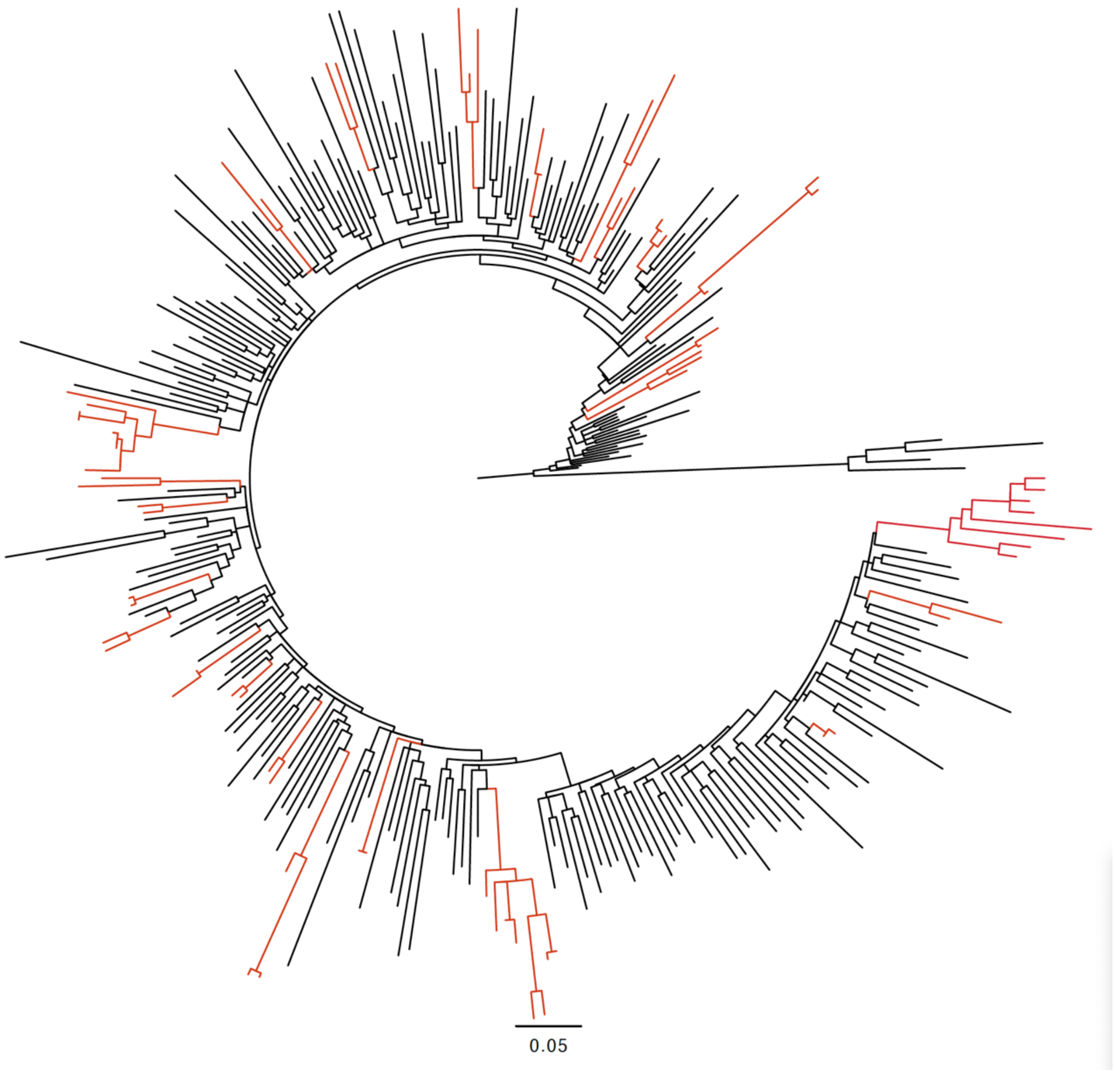

Figure S3. Phylogenetic analysis corresponding to the pol gene of 300 sequences of subtype C of HIV-1. The sequences were aligned against global control sequences from the BLAST collected from the Los Alamos database. Four reference sequences of pure subtype B were used as an outgroup. Bootstrap values equal to or higher than 90% and genetic distance less than 0.06 are represented in the tree as clusters marked in red. The scale bar indicates 5% divergence in the nucleotide sequence
